# A Compend of Human Physiology

**Published:** 1888-06

**Authors:** 


**A Compend of Human Physiology.** By ARTHUR BRUBAKER, A. M., M. D., Demonstrator of Physiology in Jefferson Medical College, etc. Fourth edition; revised and enlarged. P. Blakiston & Co. Philadelphia. 1888.

The fact that a fourth edition of this work has been called for within three years of the publication of the first, indicates that it supplies a real want among medical students. It is a true compend, and contains nearly everything the student or practitioner is expected to know about the subject. Physicians will find this book an excellent means of acquainting themselves in a short time with the great advances made in this department of medical science within the past four years.

